# Associations of plasma uromodulin and genetic variants with blood pressure responses to dietary salt interventions

**DOI:** 10.1111/jch.14347

**Published:** 2021-08-07

**Authors:** Ming‐Fei Du, Shi Yao, Ting Zou, Jian‐Jun Mu, Xiao‐Yu Zhang, Gui‐Lin Hu, Chao Chu, Hao Jia, Yue‐Yuan Liao, Chen Chen, Dan Wang, Qiong Ma, Yu Yan, Ke‐Ke Wang, Yue Sun, Ze‐Jiaxin Niu, Rui‐Chen Yan, Xi Zhang, Hao‐Wei Zhou, Wei‐Hua Gao, Hao Li, Chun‐Hua Li, Ke Gao, Jie Zhang, Tie‐Lin Yang, Yang Wang

**Affiliations:** ^1^ Department of Cardiovascular Medicine First Affiliated Hospital of Xi'an Jiaotong University Xi'an China; ^2^ Key Laboratory of Molecular Cardiology of Shaanxi Province Xi'an China; ^3^ Key Laboratory of Biomedical Information Engineering of Ministry of Education Biomedical Informatics & Genomics Center School of Life Science and Technology Xi'an Jiaotong University Xi'an China; ^4^ Department of Cardiology Xi'an No.1 Hospital Xi'an China; ^5^ Department of Critical Care Medicine First Affiliated Hospital of Xi'an Jiaotong University Xi'an China; ^6^ Department of Ophthalmology Xi’an People’s Hospital Xi'an China; ^7^ Department of Cardiology Xi’an People’s Hospital Xi'an China

**Keywords:** gene polymorphism, hypertension, salt, salt sensitivity, uromodulin

## Abstract

Uromodulin, also named Tamm Horsfall protein, have been associated with renal function and sodium homeostasis regulation. The authors sought to examine the effects of salt intake on plasma and urinary uromodulin levels and the association of its genetic variants with salt sensitivity in Chinese adults. Eighty patients from our natural population cohort were maintained sequentially either on a usual diet for 3 days, a low‐salt diet (3.0 g) for 7 days, and a high‐salt diet (18.0 g) for an additional 7 days. In addition, the authors studied 514 patients of the Baoji Salt‐Sensitive Study, recruited from 124 families who received the same salt intake intervention, and investigated the association of genetic variations in uromodulin gene with salt sensitivity. Plasma uromodulin levels were significantly lower on a high‐salt diet than on a baseline diet (28.3 ± 4.5 *vs*. 54.9 ± 8.8 ng/ml). Daily urinary excretions of uromodulin were significantly decreased on a high‐salt diet than on a low‐salt diet (28.7 ± 6.7 *vs*. 157.2 ± 21.7 ng/ml). SNPs rs7193058 and rs4997081 were associated with the diastolic blood pressure (DBP), mean arterial pressure (MAP) responses to the high‐salt diet. In addition, several SNPs in the uromodulin gene were significantly associated with pulse pressure (PP) response to the low‐salt intervention. This study shows that dietary salt intake affects plasma and urinary uromodulin levels and that uromodulin may play a role in the pathophysiological process of salt sensitivity in the Chinese populations.

## 1 INTRODUCTION

Hypertension is one of the leading public health challenges worldwide because of its high prevalence and its strongly associated risks of cardiovascular disease with vascular and overall mortality.^1^, ^2^ Essential hypertension is a complex disease with a combination of genetic and environmental factors. Salt is one of the important environmental factors and excess dietary salt intake represents a predominant cause of hypertension. However, individual blood pressure (BP) response to salt load or salt restriction is heterogeneous,^3^, ^4^, ^5^ which physiological phenomenon is salt sensitivity. We and others, have shown that salt sensitivity is significantly associated with the development of end‐organ damage from hypertension including atherosclerosis, endothelial dysfunction, and renal injury.^6^, ^7^, ^8^, ^9^, ^10^ Salt sensitivity is present in a substantial proportion (25%) of the normotensive population and more commonly observed in the population with hypertension, in whom at least 50% can be detected.^11^ Previous studies showed that the importance of sodium (Na^+^) homeostasis in hypertension and salt sensitivity is well established.^12^, ^13^

Uromodulin is a 95 kDa glycoprotein, also known as Tamm‐Horsfall protein, is encoded by the *UMOD* gene located on chromosome 16p12.3.^14^, ^15^ It is exclusively synthesized by the thick ascending limb (TAL) and early distal convoluted tubule in the kidney.^16^ The larger proportion of uromodulin is secreted into the urinary tract, where it is the most abundant protein under physiological conditions and exerts anti‐inflammatory, anti‐infective, and electrolyte‐handling effects.^17^, ^18^, ^19^, ^20^ A few studies have shown that high sodium intake increases the expression and urinary excretion of uromodulin.^21^, ^22^ However, no study has studied the relationship between dietary salt intake and blood uromodulin levels. There is experimental evidence that uromodulin is linked to the pathogenesis of salt‐sensitive hypertension.^23^, ^24^ Uromodulin overexpression in transgenic mice causes abnormal activation of Na‐K‐2Cl cotransporter (NKCC2) and salt‐sensitive hypertension.^24^ However, the role of uromodulin in the development of salt sensitivity of BP in humans has not been studied previously.

In this study we examined the effects of salt intake on plasma and urinary uromodulin levels prospectively in a well‐defined cohort of Chinese patients. In addition, we assessed the association of genetic variants in *UMOD* gene with BP responses to strict dietary salt intervention in our family‐based cohort.

## 2 METHODS

The entire study consisted of two parts: (1) an interventional trial to study the effects of dietary salt intake on plasma and urinary uromodulin levels; and (2) a family‐based cohort study to examine the association of *UMOD* gene with salt sensitivity.

### 2.1 Protocol 1: An interventional trial to study the effects of dietary salt intake on plasma and urinary uromodulin levels

In order to examine the effects of dietary salt intake on plasma and urinary uromodulin levels, a total of 80 natural individuals with similar dietary habits were recruited from two villages in Liquan and Lantian Counties, Shaanxi Province, China. Details of the study design have been published elsewhere.^25^, ^26^ All patients received the chronic salt intake intervention, which was performed as previously described.^25^, ^26^, ^27^, ^28^, ^29^ Briefly, the first phase of the intervention consisted of a 3‐day baseline observation period during which participants were given a questionnaire and physical examination was performed. This was followed by a 7‐day period of a low‐salt diet (3 g of sodium chloride or 51.3 mmol of sodium per day). Finally, a high‐salt diet (18 g of sodium chloride or 307.8 mmol of sodium per day) was given for 7 days. Dietary potassium intake remained unchanged during the low‐salt and high‐salt intervention phases. To ensure compliance, the patients were required to have their breakfast, lunch, and dinner at the study kitchen during the entire study period.

This protocol was approved by the Ethics Committee of the First Affiliated Hospital of Xi'an Jiaotong University according to the Declaration of Helsinki (2008) of the World Medical Association. All participants provided written informed consent. The trial registration number was NCT02915315 (http://www.clinicaltrials.gov).

### 2.2 Protocol 2: A family‐based cohort study to examine the association of *UMOD* gene with salt sensitivity

To examine the relationship of *UMOD* gene with salt sensitivity, we applied linkage analysis to the data from the cohort of the Baoji Salt‐Sensitive Study. The cohort originally was assembled from April to November in 2004 and consisted of 514 Han adults from 124 families from seven villages in Baoji city, Shaanxi Province, China. Probands with a mean systolic blood pressure (SBP) of 130–160 mm Hg and/or diastolic blood pressure (DBP) of 85–100 mm Hg and no use of antihypertensive medications were identified by community‐based BP screening among all adults with 18–60 years of age. Both two‐generation (probands, their parents, and siblings) and three‐generation (spouses and offspring of probands) families were recruited for the study. The detailed study design has been published previously.^30^, ^31^, ^32^, ^33^ Probands, their siblings, spouses, and offspring participated in the chronic salt intake intervention in 2004, which was similar to **Protocol 1**.

The study protocol was approved by the Academic Committee of the First Affiliated Hospital of Xi'an Jiaotong University (code: 2015–128) and was clinically registered (NCT02734472). All participants in this study signed informed consent forms.

### 2.3 BP measurement and definition of blood pressure response to dietary intervention

Blood Pressure was measured by certified observers in the sitting position using a standard mercury sphygmomanometer, as previously described.^26^, ^30^, ^33^ BP measurements were performed during the 3‐day baseline observation period and on days 5, 6, and 7 of each of the two 7‐day intervention periods, respectively. The mean arterial pressure (MAP) was calculated as DBP + [1/3 × (SBP–DBP)]. Pulse pressure (PP) was calculated as SBP–DBP. BP responses were defined as follows: BP response to low salt = BP on low‐salt diet – BP at baseline; BP response to high salt = BP on high‐salt diet – BP on low‐salt diet.^29^, ^30^, ^31^, ^32^, ^33^, ^34^

### 2.4 24 h urinary biochemical analyses

24‐h urine samples were collected at baseline and on days 6 and 7 of each intervention period and frozen at −40°C. All urine samples were shipped in ambient packaging with the use of ice boxes to the clinical laboratory at the First Affiliated Hospital of Xi'an Jiaotong University in Xi'an, China. Urinary uromodulin levels were analyzed by using commercially available enzyme‐linked immunosorbent assay (ELISA) kits (Cusabio Biotech, Wuhan, China). Urinary sodium and potassium were measured by an automatic biochemical analyzer (Hitachi, Ltd., Japan) at a certified clinical laboratory. The 24‐h urinary excretions of sodium, potassium and uromodulin were calculated by multiplying the concentration of sodium, potassium and uromodulin respectively, by the 24‐h total urine volume.

### 2.5 Blood biochemical analyses

Blood samples were obtained by peripheral venous puncture on the last day of each intervention period, immediately centrifuged at 3000 × g for 10 min, and stored at –80°C until analysis. Total cholesterol, triglycerides, low‐density lipoprotein (LDL), high‐density lipoprotein (HDL), serum uric acid, alanine aminotransferase (ALT), aspartate aminotransferase (AST), serum Na^+^ and K^+^, serum creatinine, and fasting glucose levels were measured using an automatic biochemical analyzer (Hitachi, Tokyo, Japan). Details of these assays were described previously.^35^, ^36^, ^37^ Plasma uromodulin levels were analyzed by using commercially available ELISA kits (Cusabio Biotech, Wuhan, China). Intra‐assay and inter‐assay coefficient variations were 2.1–5.2% and 3.8–6.5%, respectively.

### 2.6 SNP selection and genotyping

Thirteen tagged SNPs in the *UMOD* gene (rs77875418, rs4293393, rs7193058 rs4997081, rs11859916, rs13333226, rs79245268, rs4632135, rs4383153, rs12708631, rs7198000, rs6497476 and rs12917707) were selected using the National Center for Biotechnology Information database (http://www.ncbi.nlm.nih.gov/projects/SNP) and the Genome Variation Server database (http://gvs.gs.washington.edu/GVS147/). The selection criteria for SNPs were as follows: tag SNPs in the CHB and Asian database selected by the Haploview 4.2 software (Broad Institute, Cambridge, MA, USA) with Hardy‐Weinberg equilibrium (HWE) *p*‐value ≥ .05; a minor allele frequency (MAF) ≥ 0.05; and r^2^ ≥ 0.8. All the genotyping experiments were done by CapitalBio (CapitalBio Corp, Beijing, China) as previously described.^29^, ^30^, ^31^, ^32^, ^33^

## 3 STATISTICAL ANALYSES

For the analyses in the interventional trial, differences in repeated measures were analyzed by the repeated‐measures analysis of variance. The partial correlation coefficient was measured to assess the relationship between two variables, adjusting for traditional cardiovascular risk factors and potential confounders. Statistical analyses were performed in SPSS for Windows, Version 16.0 (SPSS Inc., Chicago, IL, USA).

The Mendelian consistency of the SNP genotype data was assessed by PLINK software (version 1.9, http://zzz.bwh.harvard.edu/plink/). We used Haploview (version 4.1, http://www.broad.mit.edu/mpg/haploview) to test Hardy–Weinberg equilibrium on parental SNP data and estimate the extent of pairwise linkage disequilibrium between SNPs. We used PLINK software to test the association of single marker with phenotypes, and three genetic models (additive, dominant, and recessive) were also assumed for each SNP analysis by mixed‐effect regression models using *lme* function in *nlme* R package. Models were adjusted for the fixed effects of age, sex and BMI, and the random effect of familial correlations. Bonferroni correction was used for adjustment of multiple testing. *p* < .05 was considered statistically significant.

## 4 RESULTS

### 4.1 Baseline characteristics of patients and effects of salt intake on BP and 24 h urinary sodium excretion in the intervention trial

All patients (*N* = 80) completed the intervention trial. **Table** **1** summarizes demographic and clinical characteristics of study participants. Sixteen patients (19.8%) had hypertension, and none of them received any medication.

**TABLE 1:** Baseline demographic and clinical characteristics

Parameters	Values
Age (year)	50.4±10.6
Sex (M/F)	28/52
Body mass index (kg/m^2^)	24.1 ±3.2
Waist circumference (cm)	85.5 ±9.3
Smoking (*n*, %)	20 (24.7)
Hypertension (*n*, %)	16 (19.8)
Pulse (beats/min)	72.7±9.8
SBP (mm Hg)	116.4±16.3
DBP (mm Hg)	75.1±9.1
MAP (mm Hg)	88.8±10.6
ALT^∗^ (U/L)	20.0 (16.5～31.2)
AST^∗^ (U/L)	22.0 (19.6～28.1)
Fasting blood glucose (mmol/L)	4.51±1.00
Total cholesterol (mmol/L)	4.29±0.94
Triglycerides^∗^ (mmol/L)	1.16 (0.85～1.55)
LDL (mmol/L)	2.38±0.72
HDL (mmol/L)	1.33±0.37
Serum creatinine (μmol/L)	54.8±8.3
Serum Na^+^ (mmol/L)	141.2±1.9
Serum Cl^−∗^ (mmol/L)	104.2 (102.6～105.4)
Serum K^+^ (mmol/L)	4.35±0.65
Plasma UA (μmol/L)	258.7±72.0

Values are means ± SD or percentages.

*Abbreviations*: SBP, systolic blood pressure; DBP, diastolic blood pressure; MAP, mean arterial pressure; ALT, alanine aminotransferase; AST, Aspartate aminotransferase; LDL, low‐density lipoprotein; HDL, high‐density lipoprotein; UA, uric acid.^∗^Expressed as median (25–75%).

BP was significantly higher on a high‐salt diet than on a low‐salt diet (*p *< .05, **Table** **2**). Not unexpectedly, urinary sodium excretion paralleled salt intake, decreasing on a low‐salt diet and increasing on a high‐salt diet (*p* < .05, **Table** **2**). These results confirmed compliance with dietary interventions.

**TABLE 2:** BP levels (mm Hg) and urinary sodium and potassium excretions (mmol/d) in the interventional study

	SBP	DBP	MAP	24 h urinary Na^+^	24 h urinary K^+^
Baseline	116.9±16.4	75.3±9.3	89.2±10.8	172.1±71.8	37.9±19.1
Low‐salt diet	112.4±12.7*	75.6±8.2	87.9±9.0	91.2±38.1*	35.8±16.3
High‐salt diet	122.0±18.3**	78.7±9.0**	93.1±11.3**	266.7±71.3**	37.5±16.2

Values are means ± SD.

*Abbreviations*: BP, blood pressure; SBP, systolic blood pressure; DBP, diastolic blood pressure; MAP, mean arterial pressure.

^*^ *p *< .05 versus baseline.

^**^ *p *< .05 versus low‐salt diet.

### 4.2 Effects of salt intake on plasma and urinary uromodulin levels

**FIGURE 1:**
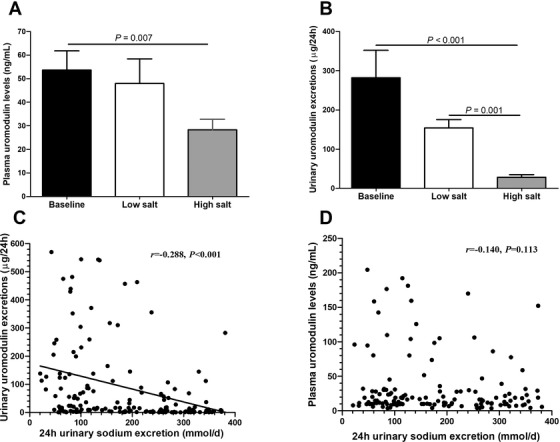
Effects of Salt Intake on Plasma and Urinary Uromodulin Levels. (A). Plasma uromodulin levels on the dietary salt interventions. (B). Daily urinary excretions of uromodulin on the dietary salt interventions. (C). The correlation between 24 h urinary sodium excretions and urinary uromodulin excretions. (D).  The correlation between 24 h urinary sodium excretions and plasma uromodulin levels

**FIGURE 2:**
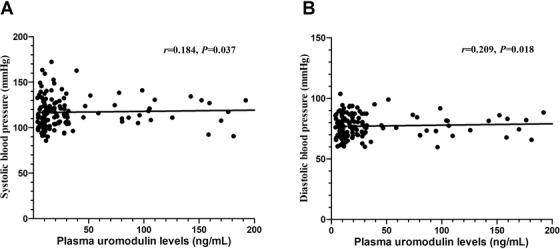
Correlations between SBP (A), DBP (B) and plasma uromodulin in all patients on a low‐salt diet and high‐salt diet

Plasma uromodulin levels were significantly lower on a high‐salt diet than on a baseline diet (28.3 ± 4.5 *vs*. 54.9 ± 8.8 ng/ml, *p* = .007) (**Figure** **1A**). In addition, daily urinary excretions of uromodulin were lower on a low‐salt diet than on a baseline diet, although not statistically significant. Interestingly, urinary uromodulin excretions were even lower on a high‐salt diet than on a low‐salt diet (28.7 ± 6.7 *vs*. 157.2 ± 21.7 ng/ml, *p* = .001) (**Figure** **1B**). The 24 h urinary sodium excretions were inversely correlated with urinary uromodulin excretions (*r* = −0.288, *p* < .001) on both low‐salt and high‐salt diets after adjusting for age, sex, body mass index, smoking, SBP, glucose, total cholesterol, serum creatinine (**Figure** **1C**), but not correlated with plasma uromodulin levels (*r* = −0.140, *p* = .113, **Figure** **1D**). In addition, plasma uromodulin levels were positively correlated with SBP (*r* = 0.184, *p* = .037, **Figure** **2A**) and DBP (*r* = 0.209, *p* = .018, **Figure** **2B**) during both low‐salt and high‐salt diet intervention periods.

### 4.3 Baseline characteristics and BP response to dietary intervention in the family‐based cohort study

**Table** **3** shows the baseline characteristics and BP responses to low‐salt and high‐salt diets in the original cohort of the Baoji Salt‐Sensitive Study (*N* = 514). Blood Pressure paralleled salt intake, decreased on a low‐salt diet and increased on a high‐salt diet. The BP changes during the dietary interventions were greater in probands than in their siblings, offspring and spouses.

**TABLE 3:** Baseline characteristics and BP response to salt intake of participants

	Probands	Siblings	Spouses	Offspring	Parents
No. of participants	99	167	18	49	181
Age (years)	41.8±8.4	39.8±7.4	47.4±6.1	23.3±6.9	66.1±8.3
Male (%)	69.7	49.1	26.3	49.0	48.4
Body mass index (kg/m^2^)	23.0±2.8	22.2±2.9	23.1±4.7	20.1±2.7	20.4±2.6
BP at baseline (mm Hg)					
SBP	120.9±12.5***	107.6±11.1	108.6±12.2	102.7±10.7	123.2±21.3
DBP	79.0±8.3***	70.1±8.1	70.6±6.9	63.4±8.9	70.5±10.5
MAP	93.0±9.0***	82.6±8.7	83.3±7.9	76.5±9.2	88.0±13.1
BP response to low salt intervention (mm Hg)					
SBP	111.7±10.0[Link], [Link]	103.4±9.1*	102.5±7.7*	100.3±9.4*	–
DBP	72.8±9.3[Link], [Link]	66.4±7.7*	67.1±5.8*	60.7±8.3*	–
MAP	85.7±9.0[Link], [Link]	78.7±7.6*	78.9±5.4*	73.9±8.3*	–
SBP change	‐8.65±9.52***	‐3.90±5.41	‐6.15±7.88	‐2.38±4.79	–
DBP change	‐6.00±6.71***	‐3.64±4.83	‐3.48±6.36	‐2.70±5.21	–
MAP change	‐6.88±7.07***	‐3.73±4.55	‐4.37±6.52	‐2.59±4.56	–
BP response to high salt intervention (mm Hg)					
SBP	118.9±11.2[Link], [Link]	108.5±11.1**	108.4±10.9**	102.0±10.0**	–
DBP	76.2±8.1[Link], [Link]	68.7±9.3**	68.6±7.5	60.9±8.3	–
MAP	90.4±8.5[Link], [Link]	82.0±9.5**	81.9±8.0**	74.6±8.4	–
SBP change	7.16±7.40***	5.09±6.50	5.93±7.90	1.72±4.07	–
DBP change	3.49±7.33***	2.29±5.73	1.51±4.69	0.22±4.52	–
MAP change	4.71±6.86***	3.22±5.60	2.98±5.61	0.72±3.79	–

Continuous variables are expressed as mean ± SD.

*Abbreviations*: BP, blood pressure; SBP, systolic blood pressure; DBP, diastolic blood pressure; MAP, mean arterial pressure.

^*^ *p* < .05 *versus* the baseline levels.

^**^ *p *< .05 *versus* the low‐salt intervention.

^***^ *p *< .05 *versus* the siblings, spouses or offspring.

As shown in **Table S1**, the urinary sodium excretion on the low‐salt diet was significantly lower than on the baseline diet and significantly higher on the high‐salt diet (*p* < .05), which indicated compliance with the dietary intervention.

### 4.4 *UMOD* and BP response to dietary intervention

The genomic location, minor allele frequency, Hardy‐Weinberg test and potential function prediction for each of the SNPs are shown in **Table** **4**. No SNP deviated significantly from Hardy‐Weinberg equilibrium.

**TABLE 4:** Information on genotyped SNPs of UMOD

SNP	Position	Region	Alleles *^a^*	MAF	*p*‐value*^b, c^*	Potential function prediction
rs4632135	20337884	intronic	T/C	0.105	1	DHS
rs4383153	20338622	intronic	G/A	0.105	1	TFBS
rs11859916	20351231	intronic	G/A	0.203	.815	DHS
rs7198000	20351937	intronic	G/A	0.193	.808	DHS
rs7193058	20360101	exonic	G/A	0.248	.384	TFBS; DHS
rs77875418	20360359	exonic	G/A	0.051	1	TFBS; DHS
rs79245268	20362115	exonic	C/T	0.051	1	DHS
rs4293393	20364588	intronic	A/G	0.057	1	DHS
rs6497476	20364781	intronic	T/C	0.051	1	TFBS
rs4997081	20365234	intronic	C/G	0.382	.079	–
rs13333226	20365654	intronic	A/G	0.057	1	–
rs12708631	20365697	intronic	T/A	0.397	.748	–
rs12917707	20381234	intronic	G/A	0.1422	.483	–

*Abbreviations*: SNP, single nucleotide polymorphism; MAF, minor allele frequency; DHS, DNase I hypersensitive sites; TFBS, Transcription factor binding site.

*^a^* Alleles are presented as major: minor allele.

^b^ *p* values of Hardy‐Weinberg equilibrium test.

^c^Parents ‐only (parental generation).

The associations of *UMOD* SNPs with the BP response to dietary intervention (after correcting for multiple testing) are displayed in **Table** **5**. SNPs rs7193058 and rs4997081 were associated with the DBP and MAP responses to the high‐salt diet. In addition, SNPs rs7198000, rs77875418, rs79245268, rs4293393, rs6497476, rs4997081 and rs13333226 were significantly associated with PP response to the low‐salt intervention.

**TABLE 5:** UMOD SNPs associated with BP response to dietary intervention

		SBP response		DBP response		MAP response		PP response	
SNP	Allele	*β*	*p*	*β*	*p*	*β*	*p*	*β*	*p*
Low‐salt intervention									
rs4632135	C	0.137	.278	0.100	.424	0.124	.321	0.078	.535
rs4383153	A	0.137	.278	0.100	.424	0.124	.321	0.078	.535
rs11859916	A	−0.011	.909	0.027	.786	0.013	.896	−0.043	.663
rs7198000	A	−0.037	.713	0.025	.809	<0.001	.997	−1.981	**.048** ^b^
rs7193058	A	−0.096	.388	0.005	.965	−0.037	.735	−0.133	.229
rs77875418	A	0.220	.186	−0.124	.454	0.011	.946	2.946	**.003** ^a^
rs79245268	T	0.220	.186	−0.124	.454	0.011	.946	2.819	**.005** ^a^
rs4293393	G	0.215	.179	−0.103	.516	0.023	.887	2.946	**.003** ^a^
rs6497476	C	0.220	.186	−0.124	.454	0.011	.946	0.424	**.011**
rs4997081	G	−0.039	.762	0.206	.111	0.118	.355	−2.050	**.005** ^a^
rs13333226	G	0.212	.184	−0.105	.509	0.020	.898	2.809	**.005** ^a^
rs12708631	A	0.057	.478	0.0002	.998	0.024	.761	0.076	.344
rs12917707	A	−0.083	.361	−0.047	.584	−0.068	.452	−0.059	.517
High‐salt intervention									
rs4632135	C	0.092	.471	0.003	.979	0.038	.766	0.122	.339
rs4383153	A	0.092	.471	0.003	.979	0.038	.766	0.122	.339
rs11859916	A	0.023	.821	0.062	.533	0.052	.605	−0.045	.653
rs7198000	A	0.034	.744	0.056	.579	0.052	.611	−0.024	.819
rs7193058	A	0.195	.082	0.223	**.042**	0.229	**.038**	−0.009	.933
rs77875418	A	0.129	.447	0.201	.227	0.188	.260	−0.072	.669
rs79245268	T	0.129	.447	0.201	.227	0.188	.260	−0.072	.669
rs4293393	G	0.107	.510	0.198	.215	0.178	.268	−0.098	.545
rs6497476	C	0.129	.447	0.201	.227	0.188	.260	−0.073	.669
rs4997081	G	−0.198	.127	−0.311	**.015**	−0.291	**.023**	0.114	.381
rs13333226	G	0.107	.510	0.196	.220	0.176	.272	−0.096	.556
rs12708631	A	0.118	.148	0.151	.060	0.149	.064	−0.025	.758
rs12917707	A	−0.025	.789	0.083	.363	0.048	.602	−0.137	.140

For associations those were not significant under any model, *β* and *P* values for an additive model are listed. All genetic models are based on the minor allele of each SNP.

Statistically values are presented in bold.

*Abbreviations*: BP, blood pressure; SBP, systolic blood pressure; DBP, diastolic blood pressure; MAP, mean arterial pressure; PP, pulse pressure; SNP, single nucleotide polymorphism.

^a^ Dominant model.

^b^ Recessive model.

^c^ Additive model.

## 5 DISCUSSION

In our interventional study, we found that high‐salt intake reduced significantly plasma uromodulin levels and daily urinary excretions of uromodulin. And urinary uromodulin excretions were negatively correlated with the 24 h urinary sodium excretion. In addition, we identified several novel *UMOD* SNPs were significantly associated with the BP responses to dietary salt interventions in a family‐based cohort. Our results further provide evidence to support that uromodulin may be involved in salt sensitivity of BP.

Limited evidence suggest that uromodulin levels are regulated by salt intake.^21^, ^22^ In rats, high salt intake increased uromodulin mRNA and protein levels in kidneys.^22^ Torffvit and coworkers^21^ showed that high dietary salt intake decreased 24 h urinary excretion rate of uromodulin in 30 hypertensive patients. Individuals with higher uromodulin levels show a 20% higher 24‐h urinary sodium excretion.^38^ Ponte and coworkers^39^ also found that patients with higher urine uromodulin levels had higher 24‐h urinary sodium, potassium creatinine excretions. Urinary uromodulin excretion was correlated with urinary sodium excretion in a community‐based Chinese cohort.^20^ In the present study, a low‐salt intake marginally decreased urinary uromodulin excretions from the baseline. Similarly, urinary uromodulin excretions on a high‐salt intake were even lower than the levels found on a low salt diet. In addition, we observed a negative correlation between the 24h urinary sodium excretion and urinary uromodulin excretions in our cohort. In addition, Graham and coworkers^40^ observed an interesting phenomenon that the lower urinary uromodulin excretion associated with the G allele of rs13333226 was present only on low‐salt diet and that this association was blunted with high salt intake, indicating a possible gene‐environment interaction. Although most studies in the past have focused on urinary uromodulin, few studies have measured uromodulin in blood. The present study builds upon these previous findings by firstly evaluating the effects of salt intake on plasma uromodulin. High salt resulted in a marked decrease in circulating uromodulin levels from baseline, enhancing our knowledge of uromodulin pathophysiology and regulation. How uromodulin levels are reduced by both the low‐salt diet and high‐salt diet remains to be explored. It can be hypothesized that the decreased urinary uromodulin excretion could be due to the decreased natriuresis during the low‐salt diet. Furthermore, a high salt intake increased the urinary concentration of sodium, which in turn decreased the urinary concentration of uromodulin in 24 h urine collection, an observation which was in agreement with an association between excretion of uromodulin and the absolute distal reabsorption rate of sodium.^41^ Further studies are needed to elucidate the molecular mechanism underlying this phenomenon.

Recently, uromodulin has been implicated in BP regulation and reported to be associated with hypertension. Padmanabhan and coworkers^42^ showed the minor G allele of SNP rs13333226 in *UMOD* gene promoter region is significantly associated with a lower risk of hypertension, reduced urinary uromodulin excretion, better renal function in a genome‐wide association study (GWAS). Graham and coworkers^40^ showed that uromodulin knockout (*UMOD* ^−/−^) mice had significantly lower SBP than the wild‐type mice, they were resistant to salt‐induced changes of BP, and they demonstrated a shift to the left of the pressure–natriuresis curve. Trudu and coworkers^24^ in contrast showed that *UMOD* overexpression caused a dose‐dependent increase in *UMOD* expression and excretion associated with increased BP. They also demonstrated that furosemide treatment significantly enhanced natriuresis and reduced BP levels both in the transgenic mouse.^24^ Aged *UMOD* ^−/−^ mice were markedly oliguric and unresponsive to furosemide and were hypertensive due to its failed translocation from the cytoplasm to the apical surface to partition in the lipid rafts.^43^ These studies indicate that the link between uromodulin and hypertension is sodium transport in the kidney. In addition, Bakhoum et. al.^44^ showed that baseline levels of urine uromodulin were not associated with change in SBP in response to an increase in sodium intake within the DASH‐Sodium trial. In contrast, Ponte et al.^39^ reported that there was a trend for higher SBP or DBP at higher sodium intake in the highest uromodulin strata, but an inverse association in the lowest one. The effects of urinary sodium on BP were different in low‐ and high‐uromodulin participants.^39^ These studies suggest that uromodulin may serve as an effect modifier in the salt intake and BP relationship. Future studies are needed to clarify the associations of salt, uromodulin and hypertension.

To our knowledge, this is the first study to show several novel *UMOD* SNPs were significantly associated with the BP response to changes in dietary salt intake. SNPs rs7193058 and rs4997081 were associated with the DBP and MAP responses to a high‐salt diet. Additionally, we identified seven *UMOD* variants (rs7198000, rs77875418, rs79245268, rs4293393, rs6497476, rs4997081, and rs13333226) that were significantly associated with the PP response to a low‐salt diet. SNP rs4293393 is located in the *UMOD* promoter, and transgenic mice expressing rs4293393 *UMOD* variant overexpressed uromodulin, leading to salt‐sensitive hypertension.^24^ After posteriori for the *UMOD* SNP rs4293393, hypertensive individuals homozygous for the risk allele, relative to carriers of the protective one, had significantly higher baseline mean DBP, and showed an increased response to the diuretic with significantly higher increase of natriuresis and response of BP.^24^ A GWAS identified the major allele of SNP rs4293393 is correlated with uromodulin gene expression, urinary excretion, chronic kidney disease, and the development of salt‐sensitive hypertension.^45^ According to these reports, SNP rs4293393 might be an important site for the regulation of *UMOD* gene expression and the development of salt sensitivity of BP.

Although the cause of salt‐sensitive hypertension is undoubtedly multifactorial, there is experimental evidence that links abnormalities in the uromodulin to the pathogenesis of salt‐sensitive hypertension. Aviv and coworkers^13^ reported that enhanced activity of the NKCC2 in the TAL of Henle's loop was the major factor contributing to the high prevalence of salt sensitivity in black populations. This finding correlated with the upregulation of NKCC2, with increased protein phosphorylation via the STE20/SPS1‐related proline/alanine‐rich kinase (SPAK) and the down‐regulation of the negative regulator kidney‐specific KS‐SPAK.^24^ Olinger and coworkers^23^ further demonstrated that hepsin‐mediated processing of uromodulin was critical to regulation of salt transport in the TAL. After 2 months of high salt intake, the defective processing of uromodulin in hepsin‐deficient mice leads to salt wasting and a loss of salt‐sensitivity evidenced by a shift in the relationship between Na^+^ intake and SBP. These modifications were associated with intracellular accumulation of uromodulin, endoplasmic reticulum‐stress and renal tubular injury.^23^ Such evidence points toward an interplay between salt intake, uromodulin, and salt sensitivity. Future functional studies are needed to elucidate how the identified risk loci contribute to salt sensitivity of BP at the molecular and cellular level.

The current study has several strengths. First, participation in the dietary interventions was high, and excellent compliance with the study interventions was noted, as evidenced by 24‐h urinary sodium excretions during each intervention period. Furthermore, stringent quality control procedures were employed for genotyping and data collection. We used the average of nine separate BP measures at each period, thus reducing measurement errors. However, this study also has several limitations. Firstly, BP measurement with a mercury sphygmomanometer may cause confounding effect of the white coat phenomena. Furthermore, since the 7‐day dietary intervention is relatively short, and a longer period of salt intervention is still needed to validate our results in the future. In addition, the novel findings in our study need to be replicated in other cohorts with different genetic background. Finally, due to the limited number of genotyped SNPs in the *UMOD* gene, less frequent genetic variants may have been omitted in the current study. Future research will be needed to explore their associations with salt sensitivity of BP.

In conclusions, we report for the first time that variations in dietary salt intake significantly influence plasma and urinary uromodulin levels. In addition, *UMOD* gene polymorphisms were significantly associated with BP responses to dietary salt interventions in a Chinese Han population. These results suggested that the uromodulin might be mechanistically involved in salt sensitivity of BP. This work contributes to the accumulating evidence that genomic differences regulate BP and hypertension development.

## FUNDING

This work was supported by the National Natural Science Foundation of China No. 81600327 (Yang Wang) and No. 81870319, 82070437 (Jian‐Jun Mu), Grants from China Postdoctoral Science Foundation funded project (No. 2018T111075 and 2018M631177), Special Financial Grant from Shaanxi Postdoctoral Science Foundation (2018BSHTDZZ14), Natural Science Basic Research Program of Shaanxi Province (2021JM‐257, 2021JM‐588), Institutional Foundation of the First Affiliated Hospital of Xi'an Jiaotong University No. 2019QN‐06, the Clinical Research Award of the First Affiliated Hospital of Xi'an Jiaotong University of China No. XJTU1AF‐CRF‐2019‐004 (Jian‐Jun Mu), Grants from the Major Chronic Non‐communicable Disease Prevention and Control Research Key Project of the Ministry of Science and Technology of China (2017YFC1307604 and 2016YFC1300104), Research Incubation Fund of Xi'an People's Hospital (No. FZ‐61), and Grant from Key Laboratory of Molecular Cardiology of First Affiliated Hospital of Xi'an Jiaotong University (KLMC‐2018‐06).

## CONFLICT OF INTEREST

The authors declare that there is no conflict of interest.

## Supporting information

Supplementary Information
