# Supplementary material for: Associations of plasma uromodulin and genetic variants with blood pressure responses to dietary salt interventions
Source: J Clin Hypertens (Greenwich). 2021 Aug 7;23(10):1897–906. doi: 10.1111/jch.14347 (PMC8678750; doi:10.1111/jch.14347)
Supplement: Supplementary file 1 — Supplementary Information [file JCH-23-1897-s001.doc]

**Table S1**. Effects of dietary intervention on urinary sodium and potassium excretions in the family-based cohort study

|  | **Probands** | **Siblings** | **Spouses** | **Offspring** |
| --- | --- | --- | --- | --- |
| Baseline | | | | |
| 24 h Urinary sodium, mmol | 225.1±11.6 | 213.8±16.4 | 218.3±20.8 | 205.4±23.4 |
| 24 h Urinary potassium, mmol | 36.8±10.8 | 38.1±9.13 | 39.4±12.3 | 35.8±15.7 |
| Low-salt intervention | | | | |
| 24 h Urinary sodium, mmol | 54.9±11.3 | 53.6±9.8 | 52.8±13.5 | 58.2±15.1 |
| 24 h Urinary potassium, mmol | 35.4±8.9 | 39.8±7.6 | 34.2±6.7 | 40.5±9.8 |
| High-salt intervention | | | | |
| 24 h Urinary sodium, mmol | 317.0±21.8 | 304.4±28.6 | 318.6±20.5 | 298.4±25.1 |
| 24 h Urinary potassium, mmol | 43.4±13.9 | 39.6±9.1 | 40.2±8.3 | 41.1±14.5 |

Continuous variables are expressed as mean ± SD.
